# Orthodontic Status and Orthodontic Treatment Need of 12- and 15-Year-Old Greek Adolescents: A National Pathfinder Survey

**DOI:** 10.3390/ijerph182211790

**Published:** 2021-11-10

**Authors:** Ioulia-Maria Mylonopoulou, Iosif Sifakakis, Elias Berdouses, Katerina Kavvadia, Konstantinos Arapostathis, Constantine J. Oulis

**Affiliations:** 1 Department of Orthodontics, National and Kapodistrian University of Athens, 11527 Athens, Greece; marilimyl@dent.uoa.gr; 2 Independent Researcher, 15231 Athens, Greece; elias@paedoclinic.gr; 3 Department of Paediatric Dentistry, European University of Cyprus, Nicosia 2404, Cyprus; k.kavvadia@euc.ac.cy; 4 Department of Paediatric Dentistry, Aristotle University of Thessaloniki, 54124 Thessaloniki, Greece; koarap@dent.auth.gr; 5 Department of Paediatric Dentistry, National and Kapodistrian University of Athens, 11527 Athens, Greece; cjoulis@dent.uoa.gr

**Keywords:** malocclusion, orthodontic status, orthodontic treatment need, modified IOTN

## Abstract

The purpose of this research was to assess the orthodontic status and orthodontic treatment needs of 12- and 15-year-old schoolchildren in Greece, in relation to sociodemographic factors and parental education level. A total of 1102 12-year-old children and 1131 15-year-old adolescents across Greece were assessed using the Modified Dental Health component (DHC) of the Index of Orthodontic Treatment Need (IOTN). An amount of 38.7% of 12-year-olds and 33.7% of 15-year-olds were in definite need of orthodontic treatment. The most common etiologic factors were tooth eruption and position anomalies. A higher rate of orthodontic treatment need was recorded among subjects with lower parental education level. Class I malocclusion was found in 50.9%, Class II in 38.4% and Class III in 10.8% of the total sample. A higher incidence of dental trauma was found in the 12-year-olds presenting with a Class II molar relationship and >3 mm overjet. The need for orthodontic treatment in Greece was higher, in comparison to other European countries, with one out of two children presenting a severe overjet associated with a high incidence of dental trauma. One out of three adolescents were still judged as having a need for orthodontic treatment by the age of 15.

## 1. Introduction

Malocclusion is defined as an irregularity of the teeth or a mal-relationship of the dental arches. It is not a life-threatening problem or disease, but it is considered a serious public health problem with the third-highest prevalence among oral pathologies after tooth decay and periodontal disease, and ranking third among world-wide public oral health priorities [1]. Malocclusions may have several implications, such as psychosocial problems related to impaired dentofacial aesthetics, disturbances of oral function (i.e., mastication, swallowing and speech), as well as greater susceptibility to trauma and periodontal disease [2]. A number of studies have demonstrated that malocclusion has a serious impact on the quality-of-life of individuals [3,4].

Epidemiological studies conducted in the field of dentistry worldwide demonstrate the extent and severity of oral diseases as well as the therapeutic needs of the population. The private and public health systems deliver dental care based on these reports [5]. According to the World Health Organization (WHO), all epidemiological data concerning the main oral diseases, as well as orthodontic problems and treatment need, should be subjected to periodic epidemiological survey [6,7].

Several epidemiological studies have been conducted in Greece. These surveys provide some insights into the orthodontic status of the population [8,9,10,11,12]. The most recent epidemiological study evaluating the type and frequency of malocclusions was conducted by Adamidis, however it was confined to a Greek district [13]. The prevalence of malocclusion and orthodontic treatment needs across the country has not been assessed yet.

The first epidemiological pathfinder survey carried out to assess the oral health status of the Hellenic population occurred ten years ago and covered a broad spectrum of all ages, using standardized criteria with calibrated examiners as suggested by the WHO [7] and Hellenic Dental Association [8]. In 2012–2013, a new national epidemiological pathfinder survey was carried out in order to re-assess the oral health status of the Hellenic population for 5-, 12-, 15-, 35–44, and 65–74-year-olds and compare the new findings to those from ten years prior. Standardized criteria and calibrated examiners [7,14] were used throughout this survey too, and it includes, among other parameters, the assessment of the orthodontic status of the subjects. The aim of this study is to present the orthodontic part of this survey, and to report on the prevalence of specific malocclusion signs and the orthodontic treatment needs of 12- and 15-year-old (y.o.) school children in relation to demographic factors.

## 2. Materials and Methods

The present epidemiological survey was part of the Greek national program “Assessment and Promotion of the Oral Health of the Hellenic Population”, which was carried out under the auspices of the Hellenic Dental Association in collaboration with both dental schools in Greece and funded by the National Strategic Reference Framework. A stratified cluster sample was randomly selected from schools across Greece according to WHO guidelines for national pathfinder surveys [7]. The initial sample was comprised of 1252 12 y.o. children and 1228 15 y.o. adolescents. Subjects already in orthodontic treatment (150 from the first and 97 from the second age subgroups) were excluded from the orthodontic evaluation. Finally, 2233 individuals—1102 12-year-olds and 1131 15-year-olds—were assessed for their orthodontic status and orthodontic treatment needs using the Modified Dental Health Component (DHC) of the Index of Orthodontic Treatment Need (IOTN) (Table 1). The modified DHC in the IOTN is a 2-grade scale index (no definite vs. definite need for orthodontic treatment) with no sub-categories. Combining borderline, little- and no-need groups into one group (no definite need for orthodontic treatment) simplifies the teaching and the use of the index [15,16].

Three communities of different socio-economic backgrounds were selected randomly within each of the main cities, while one urban and one rural community were selected randomly within each administrative region or island. In total, 24 sites (15 urban and 9 rural) were finally selected and around 50 subjects were examined at each site. Stratified random sampling was used to select 3 schools from each city. The selected schools were geographically dispersed and school size was also considered. If a school refused to take part in the survey, another school from the same area and of similar size was selected. Children with craniofacial abnormalities or enamel dysplasia and those who denied examination were excluded from the sample. The sample for the orthodontic evaluation included subjects in late–mixed (12 y.o.) and early–permanent (15 y.o.) dentition.

The examinations were carried out by dentists, calibrated by a reference examiner considered as the “gold standard”. The calibration procedure consisted of two sessions: (a) a theoretical, which included a presentation of malocclusion classification and the criteria of the modified IOTN. Practical exercises were included in this session using images of different orthodontic malocclusions. During clinical session (b), 10 children were examined from the trainees and the reference examiner. The results were analyzed and the disagreements discussed. The reference examiner explained the possible reasons for the disagreements and the same procedure was repeated with another 10 children. Each examiner was evaluated for intra- and inter-examiner reliability until an accepted level of specificity and sensitivity above 85% was accomplished [17].

All examinations were carried out by the calibrated examiners, assisted by trained recorders who were dentists too, in the classrooms of the selected schools while using reclining chairs, portable lights, dental mirrors, disposable explorers and metal gauges, under standardized conditions recommended by the WHO. The position of the child under examination provided comfort and good visibility for the examiner. All necessary measures of asepsis-antisepsis were taken, such as sterile instruments, disposable gloves and masks for each individual, in order to prevent transmission of infectious diseases.

General information, such as age, gender, county, urban/rural status, parental education and history of dental trauma/pain were recorded on individual charts. Parental education was assessed in years of maternal and paternal education and categorized into four levels: <6 years (primary), 9 years (secondary/high school), 12 years (secondary/lyceum) and >12 years (higher).

The following orthodontic variables were evaluated during the examination:

1. Dentition stage (primary, mixed, permanent).
2. Already in orthodontic treatment or not.
3. Cross-bite (anterior and/or posterior).
4. Occlusal relationships: canine and molar sagittal relationships (Angle’s classification). In the group ‘Class I malocclusion’, subjects presenting with canine and molar Class I relationship (permanent dentition) or neutroclusion (mixed dentition) with crowding or other dental malpositions were included. The group ‘Class II malocclusion’ consisted of subjects presenting with canine and molar Class II relationship (Divisions 1 and 2). The group ‘Class III malocclusion’ consisted of subjects presenting with canine and molar Class III relationship. Canine/molar relationship was recorded bilaterally.
5. Overjet.
6. Overbite.
7. Dental trauma (0 = no fracture, 1 = enamel fracture, 2 = enamel/dentine fracture, 3 = fracture with pulp exposure, 4 = crown discoloration, 5 = crown bonding, 6 = dental fistula).

Sample characteristics are described through tables showing absolute (N) and relative (%) frequencies. Associations between categorical variables were assessed using Pearson’s Chi-square tests. Associations between orthodontic treatment need and various demographic characteristics were assessed using multivariable logistic regression. All analyses were performed using Stata 14.2 (Stata Corp., College Station, TX, USA). The *p*-values less than 0.05 were considered statistically significant.

## 3. Results

Initially, 1252 12-year-old children were examined, of which 150 (12%) were already in orthodontic treatment. In addition, 1228 15-year-old adolescents were included in the sample, of whom 97 (7.9%) were already in orthodontic treatment (Table 2). Orthodontic patients were excluded from further analysis. A total of 2233 subjects were examined regarding orthodontic status and orthodontic treatment need with the modified IONT (Table 3).

### 3.1. Orthodontic Status

12-year-olds: A statistically significant difference was observed regarding dental age, i.e., girls were more mature (21.3% in mixed dentition) compared to boys (28.3%) (*p* = 0.007) (Table 4). A Class I relationship was recorded in 49.4% of children, while 41.4% presented with Angle Class II and only 9.2% with a Class III relationship. Cross-bite was found in 11.4% of the sample with the most frequent being the unilateral posterior cross-bite (8.7%). Normal overjet was found in 51.3% and 46.4% had normal overbite, while 47.7% presented with increased overjet and 52.3% with increased overbite (Table 5).

Increased incidence of dental trauma was found in subjects with a Class II molar relationship and increased overjet. Increased overjet (>3 mm) was recorded in 59% of the children with history of dental trauma (*p* = 0.019).

15-year-olds: Most of the adolescents were in permanent dentition by the age of 15. Only 3.3% of the girls and 3.8% of the boys presented with a mixed dentition (Table 6). A Class I relationship was recorded in 52.3% of this age group, Class II in 35.3% and Class III in 12.3%. Cross-bite was recorded in 8.9% and was most commonly posterior unilateral cross-bite (6.1%). Angle molar relationship and overjet were not found to have a significant relationship with dental trauma in this subgroup. An amount of 65.7% had normal overjet and 33.7% presented with increased overjet. Overbite was normal in 60.9% of the children and was increased in 37.5% (Table 5).

### 3.2. Orthodontic Treatment Need

12-year-olds: A definite need for orthodontic treatment was recorded in 38.7% of this subgroup. The highest value (58.5%) was recorded in North Greece and only 29.8% was recorded in the capital (Athens). No statistically significant differences were found between genders, urban and rural populations, and by parental education level (Table 7). The most common determinants for treatment need were tooth eruption and tooth position anomalies (Table 8). Girls had higher probabilities of needing orthodontic treatment compared to boys (Odds Ratio = 1.23) but the difference was marginally not significant (*p* = 0.076). Higher (University) father’s educational level was associated with significantly lower probabilities of orthodontic treatment need compared to lower (High School-Lyceum) (Odds Ratio 0.59; *p* =0.024) (Table 9).

15-year-olds: The highest value in the orthodontic treatment need index (54%) was recorded in North Greece. No statistically significant difference was detected between urban and rural populations. Overall, a need for orthodontic treatment was recorded in 33.7% of this subgroup (Table 7). Adolescents were found in greater orthodontic treatment need when the father and mother were of low-level education (39.1%, 38.9% respectively). In higher levels of parental education these rates decreased (25.8%, 24.7% respectively). Again, the most common determinants for treatment need were tooth eruption and position anomalies (Table 8). Girls had significantly lower probabilities of needing orthodontic treatment compared to boys (Odds Ratio = 0.73; *p* = 0.027). Higher (university) mother’s educational level was associated with significantly lower probabilities of orthodontic treatment need compared to lower (up to Junior High school) (Odds Ratio 0.60; *p* = 0.025). A similar, but marginally non-significant association was observed regarding father’s educational level (Odds Ratio 0.66; *p* = 0.066) (Table 10).

## 4. Discussion

This was the first time in Greece that an extended assessment regarding orthodontic status and orthodontic treatment needs of 12- and 15-year-olds was included in a national survey with standardized criteria and calibrated examiners. Overall, the need for orthodontic treatment was recorded in 33.7% of the 15-year-olds, a lower rate than in 12-year-olds. A lower percentage of active orthodontic treatments were recorded in the 15-year-olds (7.9%). This finding may be due to the greater rate of 12 y.o children already undergoing treatment (12%) in contrast with 15 y.o. children already in treatment (7.9%). The rate of adolescents undergoing orthodontic treatment during examination was affected only by demographic parameters (age). The highest need for orthodontic treatment for both subgroups was recorded in North Greece (58.5% and 45.6% respectively). The prevalence of cross-bite rate in the older subgroup was lower, since the correction of the transverse orthodontic anomalies is usually accomplished at an earlier growth stage.

A rather large, representative sample from all Greek regions was included and the results provide baseline data for comparison both with other European countries and internationally, as well as for planning orthodontic services in the national public health system. Initial epidemiological studies in this area have shown comparable results regarding the prevalence of malocclusion (24–27%) [8,9]. However, the distribution of these samples was not weighted, and they didn’t assess the orthodontic treatment need.

In the present study, the need for orthodontic treatment was assessed by the modified DHC of IOTN. A sum of 36.2% of the total sample (12- and 15-year-olds) was in need of orthodontic treatment. The same component of IOTN was used in several studies, revealing similar needs in the Middle East and Latin America: Turkey (38.8%) [18], Jordan (34%) [19], Iran (36.1%) [20], Brazil (34.2%) [21] and others. However, lower index scores were recorded in Europe: Italy (27.3%) [22], France (21.3%) [23] and other countries. The DHC of IOTN evaluates the signs of malocclusion and is a valuable tool in determining the treatment need priority for effective resource use in orthodontic care [15]. It is based on the view that the more a deviation differs from a given norm (the ideal occlusion), the greater the risks of future objective functional deficits or oral health problems. The modified DHC of IONT shows great discrimination ability and is suitable if the level of definite need for orthodontic treatment is most relevant. This modification was chosen in the present study for convenience reasons too, since the orthodontic evaluation was part of a wide dental survey [24]. A recent survey conducted in the United Kingdom used the modified IOTN and reported lower scores (35% of 12- and 21% of 15-year-olds in definite need vs. 38.7% and 33.7% respectively in the present study). This difference may be partly attributed to the different numbers of children undergoing treatment at the time of the survey (8% of 12- and 14% of 15-year-olds in active treatment vs. 12% and 7.9% respectively in the present study) [25].

The IOTN index has been criticized due to the inherent subjectivity of this evaluation system if used by non-orthodontists in oral health surveys alongside caries and periodontal indices [26]. A calibration exercise in an epidemiological study found inadequate agreement when using IOTN, with only a minority of examiners able to achieve Kappa scores in the “moderate agreement” range [27]. A recently conducted path model study suggested that orthodontic treatment need assessments should be based on the consequences of malocclusions for the individual, rather than their signs [26]. Moreover, only the most severe occlusal traits are considered for this categorization, despite the fact that other severe symptoms may be present [24].

The Aesthetic Component (AC) of IOTN was not used in the present study. AC is considered by some authors to be an unnecessary part of the IOTN when appraising mixed dentitions, possibly because this grading depicts only permanent dentitions. Moreover, assessing aesthetic impairment is complex and difficult to measure [27]. In both age subgroups, the two most common occlusal traits responsible for the final DHC categorization were tooth eruption and position anomalies. These observations have public dental health implications because they are associated with occlusal problems and poor periodontal conditions [28].

An Angle Class I malocclusion was recorded in 50.9%, Class II in 38.4% and Class III in 10.8% of the total sample. Anterior cross-bite was less common (2.4%) than posterior cross-bite (10.5%). Previous studies reported increased overjet as the most common trait, followed by crowding [29,30,31]. This difference may be attributed to ethnic differences. The prevalence of increased overjet and Class II is higher in Caucasians and Europeans and lower in Africans, as Class III is higher among Mongoloids. Posterior cross-bite was more prevalent in permanent dentition in Europe [32,33].

In addition, a history of dental trauma was most commonly recorded in children with a Class II molar relationship and increased overjet. This finding agrees with several recent studies, concluding that subjects with increased overjet may be at higher risk of incisal trauma [34]. Early oral health education for families is required to increase awareness of the importance of preventive measures for eliminating the serious consequences of the trauma. These measures may include a first phase of orthodontic treatment and the use of preventive mouth guards.

Low parental education level and low income are important determinants for not seeking early-age orthodontic treatment as well as for greater orthodontic treatment need in adolescence. Low parental education also appears as one of the most influential indicators for high caries level, periodontal diseases and poor oral hygiene in children and adolescents [35,36]. In the present study, parental education status was significantly associated with orthodontic treatment need and the percentage of children undergoing treatment. In the 12-year-olds, a significant positive relationship was observed between maternal education level and children in active treatment. Moreover, greater orthodontic treatment need was found in the 15-year-olds when the parents were of lower educational level, whereas in the highest level of parental education the rate of orthodontic treatment need was lower.

## 5. Conclusions

The need for orthodontic treatment in Greek children was higher (36.2%) in comparison to other studies conducted in Europe and beyond.

Approximately one out of two 12 y.o. children presented with Angle Class II malocclusion, increased overjet and incidence of dental trauma to the anterior teeth.

One out of ten subjects were in active orthodontic treatment during the survey.

Orthodontic treatment need and the decision to undergo orthodontic treatment depended on parents’ education level.

## Figures and Tables

**Table 1:** The Modified Dental Health Component of IOTN [16].

Definite Need for Orthodontic Treatment:
If any one of the occlusal anomalies below is present, there is a definite need for orthodontic treatment. The acronym “MOCDO” is used as an aide memoire: Missing teeth, Overjet, Cross-bites, Displacement of contact points (crowding), Overbite
**M** Hypodontia requiring pre-restorative orthodontics or orthodontic space closure to obviate the need for a prosthesis Impeded eruption of teeth Presence of supernumerary teeth Retained deciduous teeth
**O** Increased overjet greater than 6 mm Reverse overjet greater than 3.5 mm with no masticatory or speech difficulties Reverse overjet greater than 1 mm but less than 3.5 mm with recorded masticatory and speech difficulties
**C** Anterior or posterior cross-bites with greater than 2 mm discrepancy between retruded contact position and intercuspal position
**D** Contact point displacements greater than 4 mm
**O** Lateral or anterior open bites greater than 4 mm Deep overbite with gingival or palatal trauma

**Table 2:** Subjects in active orthodontic treatment by age.

Active Orthodontic Treatment	12 y.o.	15 y.o.
No	1102	1131
Yes	150	97
Total	1252	1228

**Table 3:** Descriptive characteristics of the population.

	12 y.o.	15 y.o.
**Gender**		
Male	554 (50.3)	532 (47.0)
Female	541 (49.1)	599 (53.0)
NA	7 (0.6)	
**Educational level—father**		
Junior High school	259 (23.5)	256 (22.6)
High School-Lyceum	508 (46.1)	499 (44.1)
University	330 (29.9)	372 (32.9)
NA	5 (0.5)	4 (0.4)
**Educational level—mother**		
Junior High school	220 (20.0)	198 (17.5)
High School-Lyceum	533 (48.4)	543 (48.0)
University	344 (31.2)	388 (34.3)
NA	5 (0.5)	2 (0.2)
**Population**		
Urban	724 (65.7)	726 (64.2)
Rural	378 (34.3)	405 (35.8)
Total	1102 (100.0)	1131 (100.0)

**Table 4:** Distribution of dentition stage between genders in 12-year-olds (excluding 7 subjects of unknown gender).

	Dentition Stage		
	Mixed N (%)	Permanent N (%)	Total N (%)
Male	157 (28.3)	397 (71.7)	554 (50.6)
Female	115 (21.3)	426 (78.7)	541 (49.4)
Overall	272 (24.8)	823 (74.6)	1095 (100.0)

**Table 5:** Distribution of occlusal traits by age.

	12 y.o. N (%)	15 y.o. N (%)
**Cross Bite**		
No	973 (88.3)	1026 (90.7)
Anterior	18 (1.6)	12 (1.1)
Posterior Unilateral	96 (8.7)	69 (6.1)
Posterior Bilateral	12 (1.1)	19 (1.7)
Unknown	3 (0.3)	5 (0.4)
**Angle Classification right**		
Ι	656 (59.5)	714 (63.1)
ΙΙ	374 (33.9)	308 (27.2)
ΙΙΙ	69 (6.3)	107 (9.5)
Unknown	3 (0.3)	2 (0.2)
**Angle Classification left**		
Ι	673 (61.1)	728 (64.4)
ΙΙ	351 (31.9)	297 (26.3)
ΙΙΙ	76 (6.9)	106 (9.4)
Unknown	2 (0.2)	728 (64.4)
**Orthodontic Treatment Need**		
No	676 (61.3)	750 (66.3)
Yes	426 (38.7)	381 (33.7)
**Overjet**		
(−3.5 mm. 0 mm)	11 (1.0)	4 (0.4)
(0 mm–3 mm)	565 (51.3)	743 (65.7)
(3 mm–6 mm)	457 (41.5)	343 (30.3)
>6 mm	68 (6.2)	38 (3.4)
Unknown	1 (0.1)	3 (0.3)
**Overbite**		
<0 mm	14 (1.3)	18 (1.6)
[0 mm–3 mm]	511 (46.4)	689 (60.9)
(3 mm–6 mm]	529 (48.0)	375 (33.2)
>6 mm	47 (4.3)	49 (4.3)
Unknown	1 (0.1)	
**Total**	1102 (100.0)	1131 (100.0)

**Table 6:** Distribution of dentition stage between genders in 15-year-olds.

	Dentition Stage		
	Mixed N (%)	Permanent N (%)	Total N (%)
Male	20 (3.8)	512 (96.2)	532 (47.0)
Female	20 (3.3)	579 (96.7)	599 (53.0)
Overall	40 (3.55)	1091 (96.45)	1131 (100.0)

**Table 7:** Orthodontic treatment need using the modified IONT Index.

		No %		Yes %	
		12 y.o.	15 y.o.	12 y.o.	15 y.o.
Population	Urban	62.4	68.3	37.6	31.7
	Rural	59.3	62.7	40.7	37.3
Gender	Male	63.9	63.5	36.1	36.5
	Female	58.8	68.8	41.2	31.2
Father′s Education	Junior High school	59.1	60.9	40.9	39.1
	High School-Lyceum	60.2	63.3	39.8	36.7
	University	65.2	74.2	34.8	25.8
Mother′s Education	Junior High school	63.6	61.1	36.4	38.9
	High School-Lyceum	60.8	61.7	39.2	38.3
	University	61	75.3	39	24.7

**Table 8:** Distribution of occlusal traits according to the modified IOTN among the 807 adolescents in need of orthodontic treatment.

	12 y.o.			15 y.o.		
	Yes N (%)	No N (%)	Unknown	Yes N (%)	No N (%)	Unknown
Cross-bite (anterior/posterior) with shift greater than 2 mm	65 (15.3)	361 (84.7)	-	40 (10.5)	341 (89.5)	-
Missing teeth	11 (2.6)	415 (97.4)	-	21 (5.5)	360 (94.5)	-
Tooth eruption anomaly	213 (50.0)	212 (49.8)	1 (0.2)	184 (48.3)	197 (51.7)	-
Overjet greater than 6 mm	71 (16.7)	354 (83.1)	1 (0.2)	40 (10.5)	339 (89.0)	2 (0.5)
Reverse overjet greater than −3.5 mm	-	425 (99.8)	1 (0.2)	-	379 (99.5)	2 (0.5)
Reverse overjet −1 to −3.5 mm	3 (0.7)	421 (98.8)	2 (0.5)	4 (1.0)	375 (98.4)	2 (0.5)
Increased overbite	53 (12.4)	371 (87.1)	2 (0.5)	33 (8.7)	347 (91.1)	1 (0.3)
Open bite (anterior/lateral)	27 (6.3)	399 (93.7)	-	28 (7.3)	353 (92.7)	-
Tooth position anomaly	164 (38.5)	262 (61.5)	-	151 (39.6)	230 (60.4)	-

**Table 9:** Association between need for orthodontic treatment and childrens’ demographic characteristics (12-y.o.). Results from a multivariable logistic regression model.

Characteristic	Odds Ratio	95% C.I.	*p*-Value
**Gender**			
Male	1		
Female	1.23	(0.98. 1.54)	0.076
**Population**			
Urban	1		
Rural	1.3	(0.95. 1.78)	0.106
**Educational level-father**			
Junior High school	1		
High School-Lyceum	0.84	(0.62. 1.14)	0.254
University	0.59	(0.38. 0.93)	0.024
**Educational level-mother**			
Junior High school	1		
High School-Lyceum	1.10	(0.80. 1.51)	0.571
University	1.21	(0.75. 1.98)	0.434

Baseline category; results adjusted also for districts (not shown).

**Table 10:** Association between need for orthodontic treatment and children’s demographic characteristics (15-y.o.). Results from a multivariable logistic regression model.

Characteristic	Odds Ratio	95% C.I.	*p*-Value
**Gender**			
Male	1		
Female	0.73	(0.56. 0.97)	0.027
**Population**			
Urban	1		
Rural	1.26	(0.93. 1.70)	0.131
**Educational level-father**			
Junior High school	1		
High School-Lyceum	0.95	(0.67. 1.34)	0.766
University	0.66	(0.43. 1.03)	0.066
**Educational level-mother**			
Junior High school	1		
High School-Lyceum	0.97	(0.67. 1.40)	0.855
University	0.60	(0.38. 0.94)	0.025

## Data Availability

The data presented in this study are available on request from the corresponding author.
